# Delayed Hypoparathyroidism following Thyroidectomy, a Diagnostic Conundrum: A Report of Three Cases from Sri Lanka

**DOI:** 10.1155/2020/1735351

**Published:** 2020-09-17

**Authors:** Piyumi Sachindra Alwis Wijewickrama, Henry N. Rajaratnam

**Affiliations:** ^1^Endocrinology Unit, National Hospital of Sri Lanka, Colombo 10, 00700, Sri Lanka; ^2^Nawaloka Hospitals PLC, 23, Deshamanya H. K Dharmadasa Mawatha, Colombo 2, 00200, Sri Lanka

## Abstract

**Introduction:**

Hypoparathyroidism, which is a common complication following total thyroidectomy can be transient in majority and permanent in 1.5% of the patients and usually occurs secondary to an inadvertent removal of parathyroid glands, mechanical or thermal injury or disruption of the vasculature. In some patients, it is observed that symptoms of hypocalcemia can occur for the first time several years after the surgery, which is known as “delayed hypoparathyroidism.” We report three cases of delayed hypoparathyroidism from Sri Lanka, presenting several years after total thyroidectomy. *Case Presentation.* Case 1- a 60-year-old Sri Lankan woman who presented with symptomatic hypocalcemia for the first time, 30 years after total thyroidectomy for follicular thyroid carcinoma. Case 2- a 53-year-old Sri Lankan woman presenting with neuropsychiatric manifestations of hypocalcemia for the first time, 12 years after total thyroidectomy for papillary thyroid carcinoma. Case 3- a 49-year-old Sri Lankan woman developing symptoms of hypocalcemia for the first time, 11 years after completion of thyroidectomy for papillary thyroid carcinoma. All these patients were detected to have low parathyroid hormone levels, without an alternative etiology for hypoparathyroidism, hence leading to a diagnosis of delayed post-thyroidectomy hypoparathyroidism.

**Conclusion:**

Delayed hypoparathyroidism is a rare phenomenon, which is secondary to progressive atrophy of parathyroid glands and slowly progressive hypovascularization of parathyroids due to scar tissue retraction following thyroidectomy. The nonspecific nature of hypocalcemic symptoms and lack of continuous follow-up for a long time after thyroidectomy could contribute to a further delay in diagnosis. However, it is an important diagnosis to consider in any patient with a history of neck surgery presenting with hypocalcemia, irrespective of the time duration of surgery, as timely diagnosis and treatment can prevent long-term complications of hypocalcemia and improve the quality of life.

## 1. Introduction

Hypoparathyroidism is the most common complication following total thyroidectomy, occurring in 7.6% following anterior neck surgeries. The majority (19–38%) of postoperative hypoparathyroidism is transient, resolving within 6 months, and permanent hypoparathyroidism is observed in 1.5% after total thyroidectomy, increasing to 6.4% in patients undergoing total thyroidectomy with prophylactic neck dissection [1]. The short half-life of parathyroid hormone (PTH) and the delicate nature of the parathyroid glands make them more prone to injury, resulting in immediate clinical manifestations shortly after or during thyroidectomy.

The underlying mechanisms for hypoparathyroidism following thyroidectomy are disruption of vascularity, thermal or electrical injury, and mechanical damage or partial/complete removal of the parathyroid glands [2]. Following surgery, a significant injury should occur before hypoparathyroidism develops, as even one normal gland is adequate to carry out normal function. Parathyroid glands are mainly supplied by the inferior thyroid artery, and the blood supply is complex, needing close attention to preserve the blood supply during thyroidectomy. Usually, hypoparathyroidism manifests immediately after surgery, and if it persists for more than 6 months, it is defined as “permanent hypoparathyroidism” [3].

Presenting symptoms of hypoparathyroidism are those of hypocalcemia, which include perioral and fingertip paresthesia, muscle stiffness and cramps, seizures, and psychiatric manifestations. Elicitable signs of hypocalcemia include the Chvostek sign, which is due to the increased irritability of the facial nerve, and the Trousseau sign, which is the carpopedal spasm secondary to induced ischemia. The Chvostek sign is known to be absent in 1/3^rd^ of patients with hypocalcemia, making it less specific than the Trousseau sign, which is present in 94% of patients with hypocalcemia [4].

Rarely, it is observed that patients can manifest features of hypoparathyroidism for the first time after several years of a thyroidectomy, which is known as “delayed hypoparathyroidism.” We report three cases of delayed hypoparathyroidism from Sri Lanka, presenting several years after total thyroidectomy.

## 2. Case presentation

### 2.1. Case 1

A 60-year-old Sri Lankan unmarried woman presented with worsening hand, feet, and perioral numbness for 6 months duration and muscle cramps of the lower limbs for 3 years. There were no seizures, headache, chest pain, or features of heart failure. She did not have features supportive of thyrotoxicosis including loose stools, sweating, or palpitations. She did not have limb weakness.

She gave a history of reduced appetite and unintended weight loss of 4 kg over 4 months. She did not have evidence of other endocrine abnormalities, including adrenal insufficiency, and there were no features supportive of an autoimmune process, to suggest a polyglandular autoimmune syndrome. She did not have chronic cough, joint symptoms, or skin rash to suggest a granulomatous or infiltrative disorder. She was consuming an average diet with adequate amounts of fish, meat, and milk and getting adequate exposure to sunlight. She did not have a history of diarrhoea or steatorrhoea or a family history of celiac disease to suggest an ongoing malabsorptive process. Her urine output was normal, and she did not have features of uremia. She was not on any drugs such as bisphosphonates or chemotherapeutic agents such as cisplatin which could lead to hypocalcemia.

She was diagnosed with follicular carcinoma of the thyroid at the age of 27 for which she underwent total thyroidectomy with lymph node excision 33 years ago. Histology revealed well-differentiated follicular carcinoma with multifocal capsular invasion and lymph node metastases. Operative notes indicate that parathyroid glands had been identified and preserved during the surgery. She was given Iodine-131 treatment once after the surgery. Her records did not reveal an immediate postoperative hypocalcemia. However, she was empirically treated with calcium and calcitriol in the immediate postoperative period for 3 months, after which it was stopped. Following surgery, she was regularly followed up and managed at an oncology unit, and there was no recurrence of the malignancy. She does not give a history of hypocalcemia for 30 years after surgery, until she developed the current symptoms 3 years back. She was on thyroxine 250 *μ*g daily.

On examination, she had a body mass index (BMI) of 18 kg/m^2^. Her mucosae were pink. There was no neck mass or lymphadenopathy. She had no dental abnormalities. She had no carpopedal spasms, and the Trousseau sign was positive, but with an absence of the Chvostek sign. Her cardiovascular system examination was normal with a pulse rate of 88 beats per minute (bpm), blood pressure of 130/80 mmHg, and without features of heart failure. Her respiratory and abdominal examinations were normal.

Her higher functions were normal, and she was oriented in time, place, and person. Her cranial nerves, upper limb, and lower limb examinations were within normal limits. There were no abnormal movements. She did not have cataracts, and the fundi were normal without papilledema. Her joint examination and the spine were normal.

Her psychiatric assessment on presentation revealed reduced sleep, lack of interest, and low mood, suggestive of depressive features, without any suicidal ideas. She did not have significant memory impairment.

At this point, after considering the history and examination, a possibility of hypocalcemia was suspected.  Table 1 summarizes her investigation results. The serum-ionized calcium was 1.1 nmol/l, in the low normal range. Serum inorganic phosphate was 4.8 mg/dl, in the upper normal limit, suggestive of hypoparathyroidism. Her intact PTH level was 12.2 pg/ml, at low normal limit. Her serum magnesium level was within the normal range. Her 25 (OH) Vitamin D level was normal at 32 ng/ml, where >30 ng/ml of Vitamin D is widely accepted to be at a sufficient range [1].  Table 1 summarizes her important investigations at the current presentation, as well as during periodic follow-ups after surgery, which indicates that the calcium and phosphate levels had been within the normal range until the current presentation.

She did not have evidence of an alternative etiology for the hypoparathyroidism, including features of autoimmunity. Thus, she was diagnosed with late onset hypoparathyroidism following thyroidectomy, manifesting 30 years after the surgery. Her thyroxine dose was reduced to 200 *μ*g daily. As she was clearly symptomatic for hypoparathyroidism, with the current calcium level, she was started on calcium carbonate, a total dose of 1500 mg daily with alphacalcidol 0.5 *μ*g three times daily, aiming to keep calcium at a low normal range. At the follow-up visit, one month after her initial presentation, her symptoms showed a remarkable improvement, and the serum-ionized calcium level had normalized to 1.2 mmol/l.

### 2.2. Case 2

A 53-year-old Sri Lankan mother of two presented with pins and needle sensations of fingers, muscle pains, and cramps for 3 months duration. She did not have perioral numbness, and there was no history of seizures or limb weakness.

She had a significant psychiatric history with low mood and lack of interest in day-to-day activities for which she was treated with fluoxetine by her psychiatrist for 3 months. Her mood disorder had responded to this treatment, but she developed paranoid delusions regarding her relatives. However, she did not have suicidal ideas or significant memory impairment. She also gave a history of fatigue, lethargy, and inertia, mainly early in the morning. Her other systemic inquiries were normal, and she did not have features of adrenal insufficiency or an ongoing autoimmune process.

She was diagnosed with papillary carcinoma of thyroid 12 years ago, at the age of 41 years, and underwent total thyroidectomy, followed by ablative Iodine-131 therapy. Since then, she had been on thyroxine 150 *μ*g/day. She did not give a history suggestive of hypocalcemia immediately after surgery and had not been on calcium or calcitriol treatment. She did not develop symptoms suggestive of hypocalcemia after surgery until the current presentation. The patient attained menopause at 50 years of age.

On examination, she was averagely built with a BMI of 23 kg/m^2^. She was not pale. She had examination features suggestive of hypothyroidism, including puffy face, dry skin, coarse hair, mild bilateral pitting ankle edema, and delayed relaxation of ankle jerks. Her Chvostek sign and Trousseau sign were negative. Her pulse rate was 64 bpm, and the blood pressure was 120/70 mmHg. Her neck examination was normal apart from the thyroidectomy scar. Neurological examination was normal. Fundi were normal without papilledema.

The initial investigations revealed Hb 11 g/dl, with normal renal functions. However, her TSH was 17.5 mIU/L and free thyroxine was 0.4 ng/dl, confirming our clinical diagnosis of hypothyroidism.

Thereafter, her thyroxine dose was optimized by gradually increasing the dose to 225 *μ*g daily over 3 months, following which her TSH was found to be 0.7 mIU/L. With this, her lethargy, inertia, and numbness of fingers improved to some extent, but still were not normalized. Her low mood also persisted, and fluoxetine dose was increased to 40 mg/day. Despite these measures, symptoms were the same even after 3 months. At this stage, the patient was reassessed and found to have silent mild bilateral cataracts. The Chvostek sign and Trousseau sign were positive at this point.

Her electrocardiogram (ECG) was normal with normal QT interval. Serum-ionized calcium was low with phosphate in the upper normal range, with high urinary excretion of calcium, suggestive of hypoparathyroidism. Her vitamin D level was within the sufficient range. The PTH level was found to be low at 7 pg/ml, confirming the diagnosis of hypoparathyroidism. Her CT scan of the brain did not reveal basal ganglia calcification.  Table 2 summarizes her investigations at periodic follow-up visits and current presentations, indicating that ionized calcium had been within the normal range in the previous visits.

She did not have evidence to suggest an alternative etiology for the hypoparathyroidism. Thus, she was diagnosed with late onset post-thyroidectomy hypoparathyroidism manifesting 12 years after surgery.

She was treated with calcium carbonate 1 g daily with calcitriol 0.25 *μ*g twice daily. After 1 month of treatment, her fatigue, lethargy, and dry skin improved and mood normalized. Ionized calcium became normal at 1.21 mmol/l, and her 24 hour urine calcium was normal.

### 2.3. Case 3

A 49-year-old Sri Lankan female presented with severe aches and pains with spasms of the hands and feet for 2 years duration. She first started to experience vague aches and pains with stiffness of shoulders 2 years ago, and she also noticed episodic cramps and spasms of the hands and feet. She did not have associated memory impairment, change in behavior, or seizures. Her appetite, weight, and bowel habits remained unchanged during this time. There was no lethargy or cold intolerance.

She had a history of toxic multinodular goiter 13 years back, for which she underwent subtotal thyroidectomy. As histology revealed papillary carcinoma of the thyroid, she had undergone completion thyroidectomy, followed by I-131 treatment. Since then, she was on long-term thyroxine replacement of 200 *μ*g daily with close follow-up and remained well until 2 years ago. She did not have records of low calcium levels after the surgery, and she had never been on calcium or calcitriol treatment until the current presentation.

On examination, she was well-looking and of average build. She had no alopecia, fungal infections, or vitiligo. The thyroidectomy scar was present, and there were no neck lumps or cervical lymphadenopathy. She did not have papilloedema or cataracts, and her upper limb and lower limb examinations were normal. She had positive Chvostek sign and Trousseau sign. Her abdominal, respiratory, and cardiovascular examinations were normal, with a normal pulse rate of 80 bpm.

Her available current and past investigations are summarized in Table 3. She had a TSH of 0.4, with normal free T4. Her serum calcium was low with marginally high serum phosphate. The PTH level was low at 9 pg/ml. Her Vitamin D was within the sufficient range.

She did not have evidence to suggest an alternative cause for hypoparathyroidism. Therefore, she was diagnosed with delayed post-thyroidectomy hypoparathyroidism with onset 11 years after the surgery.

She was started on calcium and calcitriol, and her symptoms improved with normalization of the calcium level. Currently, she is on follow-up without complications.

## 3. Discussion

This series describes 3 cases of post-thyroidectomy hypoparathyroidism, which presented several years after thyroidectomy. The main risk factors for permanent hypoparathyroidism following thyroidectomy are total thyroidectomy, advanced age at surgery, female gender, Graves' disease, and retrosternal goiter [3, 4]. All these patients were females who had undergone total thyroidectomies for thyroid malignancies. As the posterior capsule of the thyroid is usually removed during thyroidectomy for thyroid malignancy, parathyroid glands are at high risk of injury, leading to an increased risk of hypoparathyroidism in these patients [5]. Furthermore, thyroid cancer is known to have an increased risk for hypoparathyroidism when central lymph node dissection is performed, which would have added to the risk in the patient in Case 1 [3]. Risk of hypoparathyroidism after reoperation is known to be higher than the first surgery, which would have made the patient in Case 3 more vulnerable [6].

Few studies have suggested a transient hypocalcemia following radioactive iodine (RAI) treatment in high doses for thyroid cancer. One small study which enrolled 19 patients undergoing RAI treatment at 100–150 mci found that serum calcium reduced by 6 months after RAI treatment with inappropriately normal PTH levels [7]. As all these patients were treated by postoperative I-131 treatment, it could also have been a contributory factor for her hypoparathyroidism; however, there is no adequate evidence to support this hypothesis.

These patients were not on calcium or calcitriol supplements and did not give a history suggestive of hypocalcemia for 30 years, 12 years, and 11 years, respectively, following thyroidectomy. In all these cases, no clear precipitating factors of hypocalcemia could be identified and there was no evidence of alternative etiology for hypoparathyroidism such as autoimmune, granulomatous, or infiltrative disorders.

This phenomenon of delayed hypoparathyroidism is usually thought to occur secondary to progressive atrophy of the parathyroid glands. Another possible explanation is scar tissue formation in the parathyroid glands causing late onset retraction of scar tissue leading to worsening of hypovascularization and slowly progressive ischemia [8, 9]. Parathyroid vasculature undergoing arteriosclerotic changes in an elderly patient leading to infarction or atrophy of the remaining parathyroid tissue has also been described as a possible underlying mechanism for this late occurrence of the condition [10].

This can also be associated with other known causes for hypoparathyroidism, such as autoimmune hypoparathyroidism and infiltrating diseases of the parathyroid gland. As the symptoms of hypocalcemia could be nonspecific and vague, they could very well be attributed to other diseases, causing the diagnosis of hypocalcemia to be further delayed. Furthermore, it is observed that some patients can tolerate hypocalcemia for several years without severe symptoms, further delaying the diagnosis [11, 12]. These patients might not be on regular follow-up for so long after surgery, which further contributes to a late detection of this phenomenon, as seen in our patients. As the symptoms could be precipitated due to a superadded vitamin D deficiency, which is highly prevalent in Sri Lanka, it should always be looked for and corrected in these patients.

Only a few cases of delayed hypoparathyroidism following thyroidectomy have been reported in the literature. Simões et al. reported a patient with hypocalcemia presenting 33 years after a thyroidectomy, with a seizure [10]. Kamath and Rao reported a patient who developed features of hypoparathyroidism after 15 years of a thyroidectomy which was diagnosed further 10 years later when the patient presented with a seizure [11]. Halperin et al. reviewed 4 patients with delayed hypoparathyroidism who presented 5–23 years after total thyroidectomy with paresthesia, seizures, and psychiatric manifestations [12]. Bellamy and Kendall-Taylor reported a case of hypoparathyroidism presented for the first time 36 years following a total thyroidectomy for papillary thyroid carcinoma and suggested that latent hypocalcemia following thyroidectomy could be more common than it actually appears to be [13].

These cases highlight the rare occurrence of delayed hypoparathyroidism following thyroidectomy and add to the body of the existing literature regarding this poorly understood phenomenon. Moreover, it is important to consider delayed hypoparathyroidism in any patient with a history of neck surgery, presenting with mild, vague symptoms or severe symptoms of hypocalcemia, despite the time duration after the surgery, as well as to monitor the patients undergoing thyroidectomy for symptoms of hypocalcemia for several years after surgery.

## 4. Conclusions

Hypoparathyroidism following thyroidectomy is a well-known phenomenon which is transient or permanent. Late-onset hypoparathyroidism after several years of thyroidectomy is rare and occurs due to underlying progressive ischemia or atrophy of the parathyroid glands which were identified and preserved during surgery, but gives rise to hypocalcemia several years after the surgery. This diagnosis can easily be missed in these patients, as the symptoms are relatively vague and may mimic many other diseases. They might also not be regularly followed up long enough for the suspicion to be aroused. However, the correct diagnosis and treatment can effectively alleviate symptoms and prevent long-term complications of hypocalcemia. Therefore, although rare, this is an important phenomenon which clinicians should have in mind when evaluating patients with a history of neck surgery, irrespective of the timing of surgery.

## Figures and Tables

**Table 1:** Summary of investigations of case 1.

Investigation	Immediately after surgery	12 years after op	24 years after op	30 years after op	Normal range
Serum-ionized calcium	1.13 mmol/l	1.26 mmol/l	1.2 mmol/l	1.1 mmo/l	1.12–1.32 mmo/l
Serum phosphate	4.3 mg/dl	3.5 mg/dl	3.6 mg/dl	4.8 mg/dl	2.7–4.5 mg/dl
Serum potassium				4.6 mmo/l	3.5–5.1 mmol/l
Serum sodium				142 mmo/l	136–146 mmol/l
Serum magnesium				0.9 mmol/l	0.85–1.1 mmol/l
Intact PTH				12.2 pg/ml	10–69 pg/ml
S. 25(OH) vitamin D level				32 ng/ml	30–100 ng/ml
Thyroid stimulation hormone (TSH)	25 mIU/l	0.003 mIU/l	0.02 mIU/l	0.004 mIU/l	0.4–4 mIU/l
Fasting blood sugar (FBS)				88 mg/dl	

**Table 2:** Summary of investigations of case 2.

Investigation	Immediately after surgery	1 year after op	5 years after op	12 years after op- 3 months before	12 years after op- current presentation	Normal range
Serum-ionized calcium	1.1 mmol/l	1.2 mmol/l	1.2 mmol/l		0.8 mmo/l	1.12–1.32 mmo/l
Serum phosphate	4.2 mg/dl	3 mg/dl	3.2 mg/dl		4.5 mg/dl	2.7–4.5 mg/dl
Alkaline phosphatase					123 IU/L	44–147 IU/l
24 hour urinary calcium excretion					270 mg/24 hours	100–300 mg/24 hours
24 hour urine phosphate excretion					400 mg/24 hours	500–1500 mg/24 hours
Serum magnesium					0.9 mmol/l	0.85–1.1 mmol/l
Intact PTH					7 pg/ml	10–69 pg/ml
S. 25(OH) vitamin D level					38 ng/ml	30–100 ng/ml
Thyroid stimulation hormone (TSH)		0.056 mIU/L	0.9 mIU/L	17.5 mIU/L	0.7 mIU/l	0.4–4 mIU/l
Free T4 (FT4)				0.4 ng/dl		0.89–1.76 ng/dl

**Table 3:** Summary of investigations of case 3.

Investigation	Immediately after surgery	1 year after op	5 years after op	13 years after op- current presentation	Normal range
Serum-ionized calcium	1.13 mmol/l	1.2 mmol/l	1.15 mmol/l	0.8 mmo/l	1.12–1.32 mmo/l
Serum phosphate	4.3 mg/dl			4.8 mg/dl	2.7–4.5 mg/dl
Alkaline phosphatase				130 IU/L	44–147 IU/l
24 hour urinary calcium excretion				280 mg/24 hours	100–300 mg/24 hours
24 hour urine phosphate excretion				550 mg/24 hours	500–1500 mg/24 hours
Serum magnesium				1.4 meq/l	1.3–2.1 meq/l
Intact PTH				9 pg/ml	10–69 pg/ml
S. 25(OH) vitamin D level				33 ng/ml	30–100 ng/ml
Thyroid stimulation hormone (TSH)		0.04 mIU/L	0.2 mIU/L	0.4 mIU/l	0.4–4 mIU/l
Free T4 (FT4)				1.4 ng/dl	0.89–1.76 ng/dl
